# Epithelioma of the Upper End of the Œsophagus

**Published:** 1880-11

**Authors:** 


EPITHELIOMA OF THE UPPER END OF THE ŒSOPHAGUS. (Case under the care of DR. POORE.)

R. S., a charwoman, aged sixty, first attended on March 1, 1877. She was much emaciated, and stated that since Christmas she had had a difficulty in swallowing which had gradually increased. Attempts at swallowing produced fits of coughing. There was some pain between the shoulders. She had spat blood on two or three occasions. She had miscarried three times, one child alive and delicate, one stillborn. On examination, the epithelium of the tongue was patchy, thick in places and thin in others, and the pharynx was slightly congested. There was decided prominence of the larynx, and slight and doubtful enlargement of glands at angle of jaw. The larynx appeared healthy when examined by the laryngoscope, but between the arytenoid cartilages and the pharyngeal wall muco-pus was seen exuding. On passing the finger into the pharynx an obstruction was felt at the upper part of the œsophagus, and a speck of blood was found upon the tip of the finger on withdrawal. The examination caused the patient to cough, and she expectorated some blood and a small fleshy particle which, when examined under the microscope, revealed nests of epithelial cells quite characteristic of epithelial cancer. The patient was advised to become an in-patient, but on her declining to do so she was ordered a morphia linctus to ease the discomfort of the throat, carbolic-acid lozenges to counteract the fetor, and cod-liver oil in large doses. After a time she ceased to attend.

Remarks: The interest of the case consists in the certainty of the diagnosis at the first visit by means of the microscope. The appearance of the tongue and the history of the miscarriage rather pointed towards syphilis, but the discovery of the structure of epithelioma in the expectorated particle left no doubt as to the true nature of the case.

*Tumor as big as a hazel-nut, growing from the Left Ventricular Band.*—W. W., aged forty-eight, a work-house master, was sent into the hospital on the 18th of July, 1878, suffering from hoarseness and dyspnœa. He had been a healthy man, with the exception of an attack of gonorrhœa. His wife had miscarried three times, but there was no direct evidence of syphilis. The patient stated that he had been liable for some time to a relaxed sore-throat, that he became hoarse somewhat suddenly about a year ago, and that during the last month his symptoms had very much intensified. On July 25 he was seen by Dr. Poore, and subjected to a laryngoscopic examination. The dyspnœa was increased when the patient extended his neck for the purpose of laryngoscopy, so that the examination was not easy, and had to be performed with all possible quickness. A tumor was distinctly visible on the left side of the larynx at its anterior part, and situated, as was thought, below the left vocal cord; the left cord being apparently stretched over it like a shining band. The breathing was stridulous during both inspiration and expiration, and on applying the hand over the thyroid cartilage a distinct vibration could be felt during both respiratory acts, especially on the left side. There was a considerable amount of tenacious glairy sputa. All the extraordinary muscles of inspiration were used, and there was some swelling of the feet. There was no prostration, and no cyanotic tint of the face.

He was placed on large doses of iodide of potassium, on the chance that the tumor might be of syphilitic origin; but no improvement having taken place in two days, it was decided that an operation was necessary. In consultation with Mr. Marshall it was decided that no attempt to remove the tumor *per vias*

*naturales* would prove successful. The base appeared to be nearly as thick as the growth itself, and the inability of the patient to submit to more than a momentary laryngoscopic examination, together with the fact that the tumor completely closed the forepart of the glottis, would have rendered the passing of an écraseur over the growth impossible; and any attempt to do so was not thought either desirable or justifiable. It was considered, therefore, that thyrotomy would be the proper operation; and it was resolved that tracheotomy should be first performed, that the thyrotomy should be done subsequently after the lapse of some days, so as to give the patient time to recover from the shock of the first operation, and to allow of the second operation being performed with greater deliberation.

Tracheotomy was performed with all possible skill by Mr. Bond, the house surgeon, but the patient never rallied afterwards, and died in a few hours.

On post-mortem examination the mucous membrane of the larynx was found to be considerably injected, especially at the upper and left part. Attached to the left ventrical band at its forepart was a smooth, lobed, hard growth as big as a hazel-nut, and with a broad base. It was of a pinkish color, and marked on its upper surface by a white streak. This appearance had led to the idea of its being situated below the cord. A microscopical examination by Mr. Boyd, the surgical registrar, showed the growth to consist principally of blood-vessels with small, round nucleated cells between them, and some few spindle-shaped cells.

*A Cockle-shell in the Trachea.*—T. M., aged eight, was sent to the Throat Department for examination by Mr. Godlee, by whom he had been admitted to the hospital, with a view to operation. It appeared that on the 4th of July the boy was playing in the Regent's park, when a small cockle-shell, which he had picked off the path and placed in his mouth, accidentally slipped into his windpipe. The boy was suffering from some dyspnœa, with slight recession of the thorax during inspiration; both inspiration and expiration were accompanied by bronchitic râles, audible all over the chest. The boy pointed to the trachea as the seat of his trouble, the pressure on a spot about one inch above the sternal notch caused him pain. He was troubled occasionally with fits of spasmodic coughing. He was a timid, fidgety lad, and by no means a good subject for laryngoscopic observation. On July 10, after much trouble, a momentary glance of the larynx was obtained, and a white body was seen below the vocal cords. On the following morning a further examination was made, and a white body was distinctly visible on the anterior wall of the trachea, about an inch below the vocal cords. While endeavoring to demonstrate the presence of this foreign body to one of the students who was present, the patient was seized with a very violent fit of coughing, and coughed up a small cockle-shell nearly as big as a threepenny piece. The shell was perfect with sharp edges, and had apparently got fixed in the mucous folds of the trachea. The shell had been in the trachea just a week. After coughing up the shell all symptoms disappeared, and the patient left the hospital on the following day quite well.

